# Work-family-conflict in the context of the working conditions of university employees – comparison of professions

**DOI:** 10.1192/j.eurpsy.2021.1073

**Published:** 2021-08-13

**Authors:** L. Jerg-Bretzke, M. Kempf, S. Walter, H. Traue, P. Beschoner

**Affiliations:** Klinik Für Psychosomatische Medizin & Psychotherapie, Universität Ulm, Ulm, Germany

**Keywords:** professions, working conditions, compatibility, Work-family-conflict

## Abstract

**Introduction:**

Working conditions at universities are often considered precarious. Employees complain of fixed-term contracts and extensive unpaid overtime (Dorenkamp et al. 2016). Studies from various fields of work show that occupational groups with a high workload suffer particularly from a conflictual compatibility of work and family.

**Objectives:**

The aim of this study was to assess the WFC in the context of working conditions.

**Methods:**

N=844 university employees (55% women, 41% men) were asked about the burden of work/life balance using Work-family-conflict (WFC) - Family-work-conflict (FWC) -Scales (Netemeyer 1996). The dichotomously formulated question on overtime worked was supplemented by a five-step scaled item on the burden of overtime. The correlation analyses were calculated according to Spearman.

**Results:**

Overtime performed by 83% of the total sample and 64% feel burdened by it. 95% of the scientists and physicians, 68% of the administrative staff, 63% of the service providers work overtime and 90% of the physicians and 72% of the scientists feel burdened by it. Significantly high correlations were found between the burden of overtime and the conflict of compatibility. The higher the burden of overtime, the higher the WFC and FWC. The highest correlation was found among physicians (r=.649), followed by scientists (r=.533), administration (r=.451), services (r= (total sample r=.562).

**Conclusions:**

The additional work and strain caused by this, as well as the connections with the problem of compatibility, show need for action for employers regarding the working conditions of physicians and scientists. Especially with regard to reducing overtime and improving the compatibility of work and family.

